# Saint Peter and Saint Paul Archipelago barcoded: Fish diversity in the remoteness and DNA barcodes reference library for metabarcoding monitoring

**DOI:** 10.1590/1678-4685-GMB-2021-0349

**Published:** 2022-10-03

**Authors:** Marcelo Merten Cruz, Lilian Sander Hoffmann, Thales R. O. de Freitas

**Affiliations:** 1 Universidade Federal do Rio Grande do Sul, Programa de Pós-Graduação em Genética e Biologia Molecular, Departamento de Genética, Porto Alegre, RS, Brazil.

**Keywords:** Biodiversity, conservation, DNA barcoding, island, primer

## Abstract

In order to monitor the effects of anthropogenic pressures in ecosystems, molecular techniques can be used to characterize species composition. Among molecular markers capable of identifying species, the cytochrome c oxidase I (*COI*) is the most used. However, new possibilities of biodiversity profiling have become possible, in which molecular fragments of medium and short-length can now be analyzed in metabarcoding studies. Here, a survey of fishes from the Saint Peter and Saint Paul Archipelago was barcoded using the *COI* marker, which allowed the identification of 21 species. This paved the way to further investigate the fish biodiversity of the archipelago, transitioning from barcoding to metabarcoding analysis. As preparatory steps for future metabarcoding studies, the first extensive *COI* library of fishes listed for these islands was constructed and includes new data generated in this survey as well as previously available data, resulting in a final database with 9,183 sequences from 169 species and 63 families of fish. A new primer specifically designed for those fishes was tested *in silico* to amplify a region of 262 bp. The new approach should guarantee a reliable surveillance of the archipelago and can be used to generate policies that will enhance the archipelago’s protection.

## Introduction

Impacts of human-induced climate change, habitat fragmentation, and over-exploitation of natural resources have depleted global biodiversity, in particular in the marine environment (Díaz et al., 2006; Butchart et al., 2010; Pinsky et al., 2019). Conservation efforts based on robust biomonitoring programs are necessary to identify and mitigate ecological issues (Stat et al., 2017; Berry et al., 2019); therefore, preservation of diversity depends on species classification accuracy (Thomsen and Willerslev, 2015; Lin et al., 2020). The species composition and distribution can act as an environmental indicator of human activity (DiBattista et al., 2020).

Species are rapidly going extinct as a result of these anthropogenic activities, and it is impossible to describe the true magnitude of the loss with traditional monitoring approaches (Blaxter, 2003; Hubert and Hanner, 2015; Zamani et al., 2022); hence, molecular techniques have been developed to characterize species diversity quickly and reliably (Krishnamurthy and Francis, 2012; Elbrecht et al., 2019). Since the early 1990s, the mitochondrial gene cytochrome c oxidase I (*COI*) has been used as a tool to describe biodiversity (Folmer et al., 1994). The field was revolutionized when Hebert et al. (2003) proposed that the “Folmer region” of *COI* could be used to identify and discriminate species as a molecular barcode (Hebert *et al.*, 2003; Hebert and Gregory, 2005). This 658 bp genetic fragment can be easily obtained from animal tissues, and once sequenced, it provides greater than 97% confidence for differentiating species by the divergence in their *COI* sequences (Hajibabaei et al., 2005; Meusnier et al., 2008). After nearly two decades, the method has been widely accepted as the standard procedure for surveying biodiversity (Hubert and Hanner, 2015; Delrieu-Trottin et al., 2019).

However, for reliable species descriptions, DNA barcoding is not sufficient, and additional taxonomic approaches are necessary (Zamani et al., 2022). In fact, one of the major limitations of the technique is the need to have a reference library of DNA sequences that is built from morphologically identified species (Christoffer and Endre, 2005). This need for reference specimens imposes further difficulties because some species are rare or difficult to sample (Ogwang et al., 2020). This is exacerbated when sampling specimens from remote marine protected areas, which is the case of the Saint Peter and Saint Paul fishes.

**Figure 1 -:**
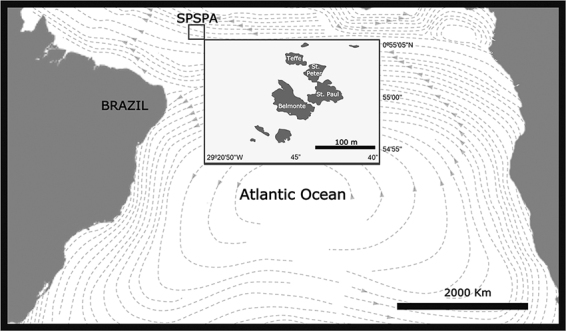
Saint Peter and Saint Paul Archipelago (SPSPA) in a map showing its geographical location (white square) in the Mid-Atlantic Ridge.

The Saint Peter and Saint Paul Archipelago (SPSPA) is a small group of plutonic rocks uplifted from the upper mantle of the earth, located in the central equatorial Atlantic Ocean between Brazil and the African continent (Figure 1; Campos et al., 2005). The archipelago is a rare non-volcanic formation resulting from the Mid-Atlantic Ridge’s exhumed mantle rocks (Mohriak, 2020). As a consequence of unique geological traits, along with latitude, weather, marine currents, and biogeographic features, the biodiversity of the SPSPA is commensurately singular. The archipelago is an important migratory, breeding, and feeding site for fishes (Mendonça et al., 2018). Also, its isolation spawned the evolution of a unique biodiversity of fishes, with a variety of color morphs and genetically divergent lineages (Pinheiro et al., 2020).

Due to this, the fish biodiversity of SPSPA has been intensively studied since the time when Lubbock and Edwards (1981) listed 50 fish species. The authors surprisingly considered the species diversity the lowest of any tropical island studied to date. Following the inauguration of the archipelago’s first scientific station in 1998, SCUBA (Self-Contained Underwater Breathing Apparatus) expeditions were made possible (Viana et al., 2009), and gradually the number of identified species increased from 75 (Feitoza et al., 2003) to 116 (Vaske Jr et al., 2005); and, most recently, to 225 species (Pinheiro et al., 2020). Contrary to Lubbock and Edwards’s (1981) considerations, the last survey pointed to the archipelago as having the third-highest level of endemism in the Atlantic (10 endemic species; Pinheiro *et al.*, 2020).

Among the 225 listed species, 112 are pelagic, 86 are shallow, and 27 are deep reef shore fishes. The inventory classification consists of 202 *Teleostei* distributed in 16 orders and 23 *Elasmobranchii* in six orders (Pinheiro et al., 2020). There are at least 29 endangered species inhabiting the SPSPA waters according to the IUCN and Brazilian Red lists (Pinheiro *et al.*, 2020). Naturally, the research collection of these species is limited by strict policies meant to protect the species; therefore, other sampling strategies are required to survey the genetic diversity of these fishes.

Fortunately, advanced molecular technologies including new DNA extraction protocols (Taberlet et al., 2018) and high-throughput sequencing have made it possible to sequence DNA molecules expelled by organisms into the environment through urine, reproductive and digestive materials, hair, skin, tissues, and decaying carcasses (Thomsen and Willerslev, 2015; Wangensteen et al., 2018). The genetic assessment of multiple taxa from bulk environmental samples is denominated “DNA metabarcoding” (Taberlet *et al.*, 2018). And now ecologists have the necessary tools to analyze the species composition of environmental samples (Taberlet *et al.*, 2012; Creer et al., 2016).

However, the genetic material extracted from ecosystems is highly fragmented (Deagle et al., 2006); to this extent, it may be challenging in practice to retrieve full-length *COI* barcode sequences (658 bp) from environmental samples (Meusnier et al., 2008). Metabarcoding analyses are contingent on targeting shorter DNA regions (<350 bp) than the traditionally defined barcoding regions (Yu et al., 2012; Clarke et al., 2014; Thomsen and Willerslev, 2015). In this context, alternative target metabarcoding markers (metabarcodes) have been developed to obtain biodiversity information in short-length (150-250 bp) PCR products (Taberlet et al., 2018).

One metabarcode option is the much shorter “*mini-COI*” barcode, a 130 bp fragment of the full ca. 658 bp *COI* barcode; Meusnier et al (2008) developed a universal primer set for the amplification of *mini-COI* that provides sufficient taxonomic resolution to differentiate between 1,587 metazoan species. Their results suggested that the region provides efficient taxonomic identification success, and its use was proposed to analyze environmental mixtures (Meusnier *et al.*, 2008); however, the mini-barcode is not variable enough to differentiate between fish species. (Sultana et al., 2018).

Medium-sized (~320 bp) barcodes that are capable of differentiating between fish species have been developed and used in marine metabarcoding studies, and to identify fish species in processed forms. (Shokralla et al., 2015; Collins et al., 2019). Despite the successful use of these markers in fish biodiversity assessment via metabarcoding (Singer et al.,2019; McClenaghan et al., 2020; Russo et al., 2021), biodiversity assessments could be maximized by the use of regional-specific reference barcode libraries (Lin et al., 2020).

In order to better characterize the baselines of Saint Peter and Saint Paul’s fish biodiversity, we collected fishes and generated full barcode sequences. For future metabarcoding monitoring of this region, we constructed a *COI* reference library of listed fish species from SPSPA, adding our sequences to those previously published. Using this library, we identified a primer pair that would be appropriate to meta-amplify fragmented *COI* barcodes of SPSPA fishes.

## Material and Methods

Five field expeditions were conducted between 2005 and 2015 in surroundings of the Saint Peter and Saint Paul Archipelago (000° 55ʼ N and 029° 21ʼ W; Fig 1). Fishes were opportunistically sampled from authorized longline catches targeting wahoos and tunas (license number SISBIO/ICMBio 014/2005). Muscle fragments were labeled (numbered) and preserved in 96% ethanol at −20°C until their extraction. Sampled fishes were identified following on-site taxonomic guides (Menezes et al., 2003).

DNA was extracted using the PureLink™ Genomic DNA Mini Kit (Thermo Fisher Scientific, Massachusetts, United States) following the manufacturer’s protocol. The forward FishF2 (5′ TCG ACT AAT CAT AAA GAT ATC GGC AC 3′) and reverse FishR2 (5′ ACT TCA GGG TGA CCG AAG AAT CAG AA 3′) primer pair (Ward et al., 2005) was used to amplify the cytochrome c oxidase I (*COI*) gene by polymerase chain reaction (PCR). Each PCR reaction was conducted in a total volume of 25 μL, consisting of 0.2 mM of dNTPs, buffer 1× 1.5 mM of MgCl2, 0.2 μM of each primer, 1 U of AmpliTaq Gold DNA polymerase (Thermo Fisher Scientific, Massachusetts, United States), 50-100 ng of template DNA quantified using NanoDrop 2000 (Thermo Scientific, Massachusetts, United States), and ultrapure water to a final volume.

The thermal cycling condition began with an initial denaturing at 94 °C for 5 minutes, followed by 35 repeated cycles of denaturing (94 °C for 0.5 minutes), annealing (50 °C for 0.5 min) and extension (72 °C for 1 min), then concluded with a final extension at 72 °C for 7 min. The size and specificity of amplification products were confirmed in 1% agarose gel stained with GelRed (Biotium, Fremont, California). The successful products were purified using exonuclease I and Shrimp Alkaline Phosphatase enzymes (Amersham Biosciences, Little Chalfont, UK). Finally, they were sequenced by the Sanger method on an ABI3730XL DNA sequencer (Thermo Fischer Scientific, Massachusetts, United States) in Macrogen Inc. (Seoul, South Korea), with the forward primer used for amplification.

The sequences were quality checked, and low-quality regions were removed by using the software Geneious Pro version 9 (Biomatters Ltd, Auckland, New Zealand). The removal of chimeric sequences and alignment using ClustalW (Edgar, 2004) were also performed in Geneious software. Species were identified using the “Identification Engine” of the Barcode of Life Data System (BOLD) by selecting ‘Animal Identification (*COI*)’ and the ‘Species Level Barcode Records’ (accessed 10 June 2021).

The taxonomic identity of each sequence was assigned to the deposited sequence with the highest similarity score. Also, a neighbor-joining tree was constructed based on the aligned dataset using the Kimura 2-Parameter (K2P) model (Kimura, 1980) with 1,000 bootstrap replicates and pairwise deletion in Geneious to cluster candidate species based on their sequences’ similarities.

As the sequenced samples represent only a small fraction of listed Saint Peter and Saint Paul fishes, the names listed in the Pinheiro et al. (2020) study were used to perform a mining within BOLD. Globally distributed *COI* sequences from the listed species were added to a new SPSPA *COI* reference database for further reference database expansion. The scientific fish names from the Pinheiro *et al.* (2020) checklist were searched on the BOLD “Taxonomy Browser” (accessed 15 June 2021). All available *COI* sequences were subsequently deposited in the SPSPA *COI* database. A detailed list of specimens and their BOLD IDs is given in Table 1. Then overall mean distance by (K2P) was computed using MEGA X software (Kumar et al., 2018).

**Table 1 -:** Sample identification, identified species, their family, similarity to the BOLD database candidate species (%), location of the BOLD matching sequence, deposited sequence (GenBank accession number), and size of the fragment. Identified fishes of Saint Peter and Saint Paul Archipelago.

Sample identification	Candidate species name (BOLD accession number)	Family	Identity (%)	Sampling location of the matching sequence	Deposited sequence (GenBank accession number)	Size of the fragment
1	*Canthidermis maculata* (LIDB123-11)	Balistidae	98.04	Belize	OK030800	540 bp
2	*Ginglymostoma cirratum* (PHANT057-08)	Ginglymostomatidae	100	United States	OK030801	515 bp
3	*Thunnus atlanticus* (MFLE487-14)	Scombridae	99.84	Honduras	OK030802	625 bp
4	*Acanthocybium solandri* (MXII111-07)	Scombridae	100	Mexico	OK030803	660 bp
5	*Coryphaena hippurus* (MXII093-07)	Coryphaenidae	100	Mexico	OK030804	606 bp
6	*Carcharhinus falciformis* (GBMND3415-21)	Carcharhinidae	100	Brazil	OK030805	629 bp
7	*Canthidermis maculata* (GBMND69325-21)	Balistidae	100	United States	OK030806	628 bp
8	*Caranx bartholomaei* (BZLWD025-07)	Carangidae	100	Belize	OK030810	625 bp
9	*Xiphias gladius* (ANGBF8490-12)	Xiphiidae	100	Not informed	OK030811	642 bp
10	*Canthidermis maculata* (FOAH793-08)	Balistidae	100	Indonesia	OK030807	630 bp
11	*Thryssa chefuensis* (ANGBF1012-12)	Coryphaenidae	100	South Korea	OK030812	625 bp
12	*Sphyrna lewini* (GBMND3593-21)	Sphyrnidae	100	Brazil	OK030813	650 bp
13	*Carcharhinus limbatus* (ANGBF48501-19)	Carcharhinidae	100	Brazil	OK030814	651 bp
14	*Acanthocybium solandri* (MXII111-07)	Scombridae	100	Mexico	OK030809	612 bp
15	*Cheilopogon atrisignis* (ANGBF32051-19)	Exocoetidae	100	Taiwan	OK030815	635 bp
16	*Remora brachyptera* (MFC279-08)	Echeneidae	100	Panama	OK030816	652 bp
17	*Sphyrna zygaena* (GBMNC59337-20)	Sphyrnidae	100	United States	OK030817	655 bp
18	*Xiphias gladius* (ANGBF36944-19)	Xiphiidae	100	Belgium	OK030818	620 bp
19	*Prionace glauca* (GBGC9258-09)	Carcharhinidae	100	Italy	OK030819	598 bp
20	*Caranx lugubris* (SABA054-11)	Carangidae	100	Saba (Caribbean Netherlands)	OK030820	633 bp
21	*Canthidermis maculata* (MEFM383-06)	Balistidae	100	Mexico	OK030808	522 bp
22	*Elagatis bipinnulata* (MXIII391-09)	Xiphiidae	100	Mexico	OK030821	620 bp
23	*Remora australis* (TZSAL697-13)	Echeneidae	100	South Africa	OK030822	627 bp
24	*Halichoeres radiatus* (BZLWA436-06)	Labridae	100	Belize	OK030823	607 bp
25	*Cheilopogon nigricans* (ANGBF32059-19)	Exocoetidae	100	Atlantic Ocean	OK030824	648 bp
26	*Prionace glauca* (GBMND3512-21)	Carcharhinidae	100	Brazil	OK030825	598 bp

A new primer pair exclusively curated (based on the physical properties, penalities of hairpin formations and primer-dimers of the SPSPA sequences database) was designed in the Primer3 plugin featured in Geneious Software (Untergasser et al., 2012). The performance of the newly designed primers was tested *in silico* against Saint Peter and Saint Paul fish sequences repository using the “Add Primers to Sequence” Geneious tool. Among the candidates’ primer pairs, the selected was the one with the highest “Pairwise Identity” targeting all the sequences of the database and with a product size appropriate for future metabarcoding studies.

## Results

**Figure 2-:**
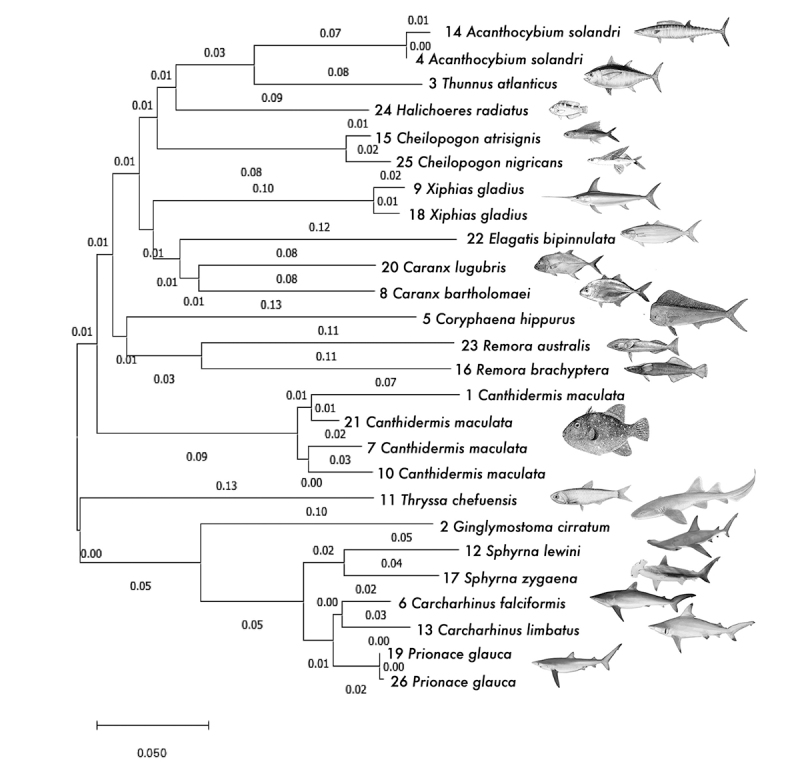
Neighbor-Joining Tree of the Saint Peter and Saint Paul Archipelago surveyed fish species labeled with substitutions *per site.*

The first attempt to barcode fishes from SPSPA waters resulted in 28 captured samples, following strict collection rules as a maximum of six fishes could be caught per expedition. The extraction, amplification, and sequencing methods were successful for 26 out of 28 samples (representing 11.55% of the known SPSPA fishes). Among the 26 samples, the *COI* Barcode could be identified on BOLD with a high percentage of similarity (98.04%-100%; Table 1), revealing 21 species that are found in 11 families of fishes (graphically represented in Figure 2). The sequences were deposited in GenBank under accession numbers OK030800-OK030825. The neighbor-joining tree revealed expected patterns - closely related species in the same genus clustered together while dissimilar species appeared on different branches. Among the 21 species of fish, *Canthidermis maculata* was the most abundant (three of the samples), followed by *Acanthocybium solandri, Xiphias gladius,* and *Prionace glauca* (two samples each). Table 1 also indicates the closest match and where the matching sequence was collected.

Of the 21 newly identified fishes, four were not listed in Pinheiro et al. (2020). Those records were then added to a new database. While 165 of the 225 species listed in Pinheiro *et al.* (2020) have *COI* sequences deposited in the BOLD database from a fish caught somewhere else, these were also used to complete the database. Therefore, the new Saint Peter and Saint Paul sequence database has 9,183 sequences from 169 species and 63 families of fish. The full reference library can be found at https://github.com/marcelomcruz4/SPSPAfishes. From this species list, 84 are pelagic, 83 are reef-associated or deep-water residents, and two are endemic (*Emblemariopsis signifier* and *Stegastes sanctipauli*)*.* The overall mean distance among all sequences was 0.4. Coherently, the AT content was higher than the GC content in the barcoded collected fishes (56.30%), and among the constructed database (AT content: 55.70%).

From this database four new primer pairs were designed. The one with the highest “Pairwise Identity” rate (74.6%) and with the most adequate target size to be amplified is presented below:

SPSPAF-5′ GCTGGAGCATCTGTTGACCT3′,

SPSPAR-5′ CTCCTCCTGCAGGGTCAAAG3′.

This marker is suited to amplify a product size of 262 base pairs from the *COI* region and performs *in silico* capacity to amplify 73.6% of Saint Peter and Saint Paul’s sequences.

## Discussion

As expected from the revised theory of island biogeography for marine fishes, the SPSPA represents an important reservoir of biological diversity and a refuge for many endemic species that have diversified on these islands through time (Pinheiro et al., 2017). Naturally, the isolation has played a crucial role in the genetic diversity and endemism of the smallest remote tropical island in the world (Luiz et al., 2015). Aside from the distance, seamounts may also have played an essential function in the marine evolution of the SPSPA. The site (as a peak of the mountain range) acted as a “stepping stone” for fishes during successive periods of sea-level changes (Ludt and Rocha, 2015; Dias et al., 2019). Also, the topography and strategic location of the area make it an important feeding and reproduction ground for several migratory pelagic species, mostly with high commercial value (Viana et al., 2015; Macena and Hazin, 2016; Pimentel et al., 2020). Our results confirm the presence of some of these species, such as the blackfin tuna *(Thunnus atlanticus),* the wahoo *(Acanthocybium solandri),* the rainbow runner *(Elagatis bipinnulata),* the flying fishes *(Cheilopogon sp.),* the silky shark (*Carcharhinus falciformis*), and the blue shark (*Prionace glauca*). Due to the heterogeneity of migrants and residents of the region, molecular techniques are a useful tool to catalog and uncover the biodiversity of SPSPA.

### DNA Barcoding advantages and limitations

DNA barcoding technology provides an efficient molecular technique for species identification to elucidate global biodiversity (Hebert et al., 2003; Krishnamurthy and Francis, 2012). The mitochondrial *COI* gene has been barcoding fish species with high efficiency (Ward et al., 2009; Ward, 2012). The marine ichthyofauna was successfully characterized in Australia (Ward *et al.*, 2005), the Antarctic (Rock et al., 2008; Mabragaña et al., 2016), Canada (Steinke et al., 2009), the Arctic (Mecklenburg et al., 2010), Japan (Zhang and Hanner, 2011), India (Lakra et al., 2011), Portugal (Costa et al., 2012), Brazil (Ribeiro et al., 2012), Germany (Knebelsberger *et al.*, 2014), Taiwan (Bingpeng et al., 2018), Indonesia (Limmon et al., 2020), Pakistan (Ghouri *et al.*, 2020), and Bangladesh (Ahmed et al., 2021).

In this unprecedented study, we successfully amplified the *COI* barcode sequences for Saint Peter and Saint Paul Archipelago fishes. The surveyed site is a remote and protected oceanic island (Soares and Lucas, 2018). This bio-blitz was the first effort to barcode representatives from the SPSPA. To this extent, the sample size is limited and for this reason, the samples of this study were opportunistically collected over different expeditions. Despite these sampling challenges, the *COI* barcoding genes of 26 fish specimens were successfully amplified and sequenced. The differentiation between species through individual *COI* barcodes validates the efficiency of *COI* barcodes for identifying marine fish species.

Even though a complete and robust identification process requires additional steps (such as diagnosable morphological characters and natural history/ecological studies), a DNA bio-scan is an extremely useful method for an initial sorting of new and known biodiversity (Zamani et al., 2022). In this way, our survey opened up the possibility of uncovering the hidden biodiversity of the archipelago.

The feasibility of gathering new species’ records for the region is sustained by the fact that the DNA barcoding revolution has hastened species discovery during the last 15 years (Cao et al., 2016; DeSalle and Goldstein, 2019; Lopez-Vaamonde et al., 2021). In turn, efforts to collect and barcode fish species from specific regions aided new fish records in other regions of the globe, such as Bangladesh, Sri Lanka, and the Bay of Bengal (Rathnasuriya et al., 2019; Ahmed et al., 2021; Sharifuzzaman et al., 2021).

The methodology applied in this study revealed four new records to the Saint Peter and Saint Paul region: *Cheilopogon atrisignis; Cheilopogon nigricans; Remora australis;* and *Thryssa chefuensis.* Considering the natural history of these species, it is plausible that *Cheilopogon nigricans* and *Remora australis* inhabit the SPSPA, as their distribution is described to be in the neighboring waters of the Atlantic Ocean (Fishbase, 2021). In fact, *Remora australis* is already photo-documented at SPSPA waters (Hoffmann et al., 2008; Wingert et al., 2021); our survey corroborates the inclusion of this species in future checklists. Whereas *Cheilopogon atrisignis* and *Thryssa chefuensis* are related to the Indian and Pacific oceans respectively (Fishbase, 2021). Additional morphometric approaches must be applied in order to confirm the presence of these species in the SPSPA. In particular, the presence of *Thryssa chefuensis* must be investigated carefully, as there are no other members of the family Engraulidae reported to the archipelago (Pinheiro et al., 2020) and DNA Barcoding has the capacity to detect alien species which invade different ecosystems (Nagarajan et al., 2020).

The identification of two species from the genus *Cheilopgon* represents new records for the site and confirms the vast diversity of flying fishes in SPSPA. It is reported that at least five species of the genus inhabit the site (Pinheiro et al., 2020); thus, the assignment of *Cheilopogon atrisignis or Cheilopogon nigricans* could be a case of misidentification due to closely related species with low differentiation between *COI* sequences. This illustrates one of the limitations of *COI* barcoding methodologies; i.e., the *COI* gene is not sufficiently variable to distinguish between some closely related species (Moritz and Cicero, 2004). To overcome this limitation and confirm species identities, more data are needed from morphological characters and/or additional genetic markers.

### Future monitoring

DNA Barcoding technical limitations prompted additional research towards the technological transition to Metabarcoding. In other words, to transition from sampling individuals (DNA Barcoding) to whole communities (DNA metabarcoding; Porter and Hajibabaei, 2020). Metabarcoding is a capture-free and non-invasive tool useful for detecting rare, elusive, controlled, protected, or threatened species (Wilcox et al., 2013; Schwentner et al., 2021). With the impossibility to sample individuals from SPSPA, metabarcoding emerges as the solution to survey and monitor SPSPA fish diversity. This approach is becoming a well-established tool for monitoring fishes not only from water samples (Miya, 2022), but also from various types of samples such as air (Lynggaard et al., 2022), sediment (Ip et al., 2021), bottom trawl fishing vessels (Maiello et al., 2022), and feces (Creer et al., 2016; Jarman et al., 2018).

Although the ability to identify and describe new species is limited using *COI* metabarcoding approaches, the amount of data generated is informative for biodiversity assessment (Taberlet et al., 2018; Meierotto et al., 2019). The collection impediment compromises the construction of a barcode reference database that optimally should be composed only of local specimens (Delrieu-Trottin et al., 2019; Lin et al., 2020). To overcome this limitation, we added to the SPSPA *COI* reference database *COI* sequences that were available on BOLD from the listed species but were collected elsewhere. As future metabarcoding steps, the constructed database, as well as the generated primer pair, must be tested in vitro, preferably with SPSPA samples and then directly with SPSPA environmental samples in a pilot study (Taberlet *et al.*, 2018). Another future perspective is the constant update of the SPSPA *COI* database, this would potentially increase the coverage of endemic species in the database, which currently only has two of the 11 listed endemic species. In this case, collected specimens in the archipelago vouchered in museums, especially the endemic ones, should be barcoded and added to the database (Ward et al., 2009).

Rather than designing primers to target all fishes (Miya et al., 2015; Collins et al., 2019), here we designed primers capable of amplifying fishes found in the target geographical region. We did this by generating an alignment of *COI* sequences for fishes known to be present in the SPSPA. Fishes are the largest group of vertebrates, and the teleost and elasmobranch species are evolutionarily distant; therefore, their genetic fingerprints are dissimilar (Nelson et al., 2016). We chose to focus on only the fishes of the SPSPA in order to increase the probability of amplification using environmental samples, thus ensuring accurate monitoring and protection.

A cocktail of primers targeting other metabarcodes such as the mitochondrial *12S* or *16S* rRNA genes (Epp et al., 2012) should be considered for a comprehensive metabarcoding study of the total fish biodiversity of the region (Collins et al., 2019).

## Conservation Considerations

Due to the presence and connectivity of key species of corals, crustaceans, mollusks, fishes, marine birds, and cetaceans, SPSPA has been protected by the Ministry of the Environment of Brazil since 1986 (Francini-Filho et al., 2018). Despite the protection, commercial fishing boats were allowed to operate in the SPSPA regularly (Viana et al., 2015). In 2018, the environmental protection of the islands and surroundings was increased by the Brazilian government (Brasil, 2018). However, the vast majority of the new areas are classified as “Areas of Sustainable Use”, where “subsistence” fisheries are specifically allowed in the management plan. In practice, commercial fishing and industrial activities by regional fishing companies are also taking place in these areas, as reported by Giglio et al. (2018). Furthermore, the habitats considered more vulnerable to high environmental impact have not received integral protection. The areas of integral protection were designated in places where these activities are already unlikely or rare (Magris and Pressey, 2018).

Fine-scale geographical and temporal studies are crucial to define boundaries and to set goals for Marine Protected Areas. Therefore, systematic data collection along time and space is necessary to understand the protected ecosystem better and promote possible zoning changes. Considering the richness of SPSPA biodiversity and its lack of protection, advanced genetics tools for monitoring ecosystems are needed. In this case, DNA metabarcoding of marine water has the potential to effectively monitor and give solid periodic information to managers and policymakers (Gold et al., 2021).

## Conclusion

The Saint Peter and Saint Paul Archipelago is a reservoir of biodiversity. The strategic location of the archipelago is an important feeding and reproductive ground for a variety of migratory fishes; likewise, it is a refuge to the third-highest fish endemism level in the Atlantic. The checklist of fishes that live in shallow and deep waters has already elucidated these outstanding patterns (Pinheiro et al., 2020); as yet the genetic signatures of SPSPA fish species have remained unknown. Thereupon, this research endeavored to barcode surveyed species of the site and catalog all deposited sequences of listed fishes in the region. Challenges and limitations of the application of DNA Barcoding methodology on SPSPA fishes reveals there is yet more diversity to be discovered. Due to this, the protection of the archipelago should be enhanced and well monitored with more robust approaches. In this case, DNA metabarcoding is an emerging tool that could assist in safeguarding SPSPA fauna; therefore, the reference library and the primer pair specifically designed to study the fishes of these islands should be considered for future metabarcoding monitoring activities.

## References

[B1] Ahmed MS, Datta SK, Saha T, Hossain Z (2021). Molecular characterization of marine and coastal fishes of
Bangladesh through DNA barcodes. Ecol Evol.

[B2] Berry TE, Saunders BJ, Coghlan ML, Stat M, Jarman S, Richardson AJ, Davies CH, Berry O, Harvey ES, Bunce M (2019). Marine environmental DNA biomonitoring reveals seasonal patterns
in biodiversity and identifies ecosystem responses to anomalous climatic
events. PLoS Genet.

[B3] Bingpeng X, Heshan L, Zhilan Z, Chunguang W, Yanguo W, Jianjun W (2018). DNA barcoding for identification of fish species in the Taiwan
Strait. PLoS One.

[B4] Blaxter M (2003). Molecular systematics: Counting angels with DNA. Nature.

[B5] Butchart SH, Walpole M, Collen B, van Strien A, Scharlemann JP, Almond RE, Baillie JE, Bomhard B, Brown C, Bruno J (2010). Global biodiversity: Indicators of recent
declines. Science.

[B6] Campos TFC, Virgens J, Srivastava NK, Petta RA, Hartmann LA, Moraes JFS, Mendes L, Silveira SRM, Winge M, Schobbenhaus C, Berbert-Bor M, Queiroz ET, Campos DA, Souza CRG, Fernandes ACS (2005). Geological and palaeontological sites of Brazil.

[B7] Cao X, Liu J, Chen J, Zheng G, Kuntner M, Agnarsson I (2016). Rapid dissemination of taxonomic discoveries based on DNA
barcoding and morphology. Sci Rep.

[B8] Christoffer S, Endre W (2005). What can biological barcoding do for marine
biology?. Mar Biol Res.

[B9] Clarke LJ, Soubrier J, Weyrich LS, Cooper A (2014). Environmental metabarcodes for insects: In silico PCR reveals
potential for taxonomic bias. Mol Ecol Resour.

[B10] Collins RA, Bakker J, Wangensteen OS, Soto AZ, Corrigan L, Sims DW, Genner MJ, Mariani S (2019). Non‐specific amplification compromises environmental DNA
metabarcoding with COI. Methods Ecol Evol.

[B11] Costa FO, Landi M, Martins R, Costa MH, Costa ME, Carneiro M, Alves MJ, Steinke D, Carvalho GR (2012). A ranking system for reference libraries of DNA barcodes:
Application to marine fish species from Portugal. PLoS One.

[B12] Creer S, Deiner K, Frey S, Porazinska D, Taberlet P, Thomas WK, Potter C, Bik HM (2016). The ecologist’s field guide to sequence‐based identification of
biodiversity. Methods Ecol Evol.

[B13] Deagle BE, Eveson JP, Jarman SN (2006). Quantification of damage in DNA recovered from highly degraded
samples-a case study on DNA in faeces. Front Zool.

[B14] Delrieu-Trottin E, Williams JT, Pitassy D, Driskell A, Hubert N, Viviani J, Cribb TH, Espiau B, Galzin R, Kulbicki M (2019). A DNA barcode reference library of French Polynesian shore
fishes. Sci Data.

[B15] DeSalle R, Goldstein P (2019). Review and interpretation of trends in DNA
barcoding. Front Ecol Evol.

[B16] Dias RM, Lima SMQ, Mendes LF, Almeida DF, Paiva PC, Britto MR (2019). Different speciation processes in a cryptobenthic reef fish from
the Western Tropical Atlantic. Hydrobiologia.

[B17] Díaz S, Fargione J, Chapin FS, Tilman D (2006). Biodiversity loss threatens human well-being. PLoS Biol.

[B18] DiBattista JD, Reimer JD, Stat M, Masucci GD, Biondi P, De Brauwer M, Wilkinson SP, Chariton AA, Bunce M (2020). Environmental DNA can act as a biodiversity barometer of
anthropogenic pressures in coastal ecosystems. Sci Rep.

[B19] Edgar RC (2004). MUSCLE: Multiple sequence alignment with high accuracy and high
throughput. Nucleic Acids Res.

[B20] Elbrecht V, Braukmann TWA, Ivanova NV, Prosser SWJ, Hajibabaei M, Wright M, Zakharov EV, Hebert PDN, Steinke D (2019). Validation of COI metabarcoding primers for terrestrial
arthropods. PeerJ.

[B21] Epp LS, Boessenkool S, Bellemain EP, Haile J, Esposito A, Riaz T, Erséus C, Gusarov VI, Edwards ME, Johnsen A (2012). New environmental metabarcodes for analysing soil DNA: Potential
for studying past and present ecosystems. Mol Ecol.

[B22] Feitoza BM, Rocha LA, Luis-Júnior OJ, Floeter SR, Gasparini JL (2003). Reef fishes of St. Paul’s Rocks: New records and notes on biology
and zoogeography. Aqua.

[B23] Folmer O, Black M, Hoeh W, Lutz R, Vrijenhoek R (1994). DNA primers for amplification of mitochondrial cytochrome c
oxidase subunit I from diverse metazoan invertebrates. Mol Mar Biol Biotechnol.

[B24] Francini-Filho RB, Ferreira CEL, Mello TJ, Prates APL, Silva VN (2018). Diagnóstico biológico e socioeconômico para a proposta de criação de uma
Área de Proteção Ambiental (APA) e um Monumento Natural Marinho (MONA) no
Arquipélago São Pedro e São Paulo.

[B25] Ghouri MZ, Ismail M, Javed MA, Khan SH, Munawar N, Umar AB Mehr-un-Nisa, Aftab SO, Amin S, Khan Z (2020). Identification of edible fish species of pakistan through DNA
barcoding. Front Mar Sci.

[B26] Giglio VJ, Pinheiro HT, Bender MG, Bonaldo RM, Lotufo LOC, Ferreira CEL, Floeter SR, Joyeux J-C, Krajewski JP, Gasparini JL (2018). Large and remote marine protected areas in the South Atlantic
Ocean are flawed and raise concerns: Comments on Soares and Lucas
(2018). Mar Policy.

[B27] Gold Z, Sprague J, Kushner DJ, Marin EZ, Barber PH (2021). eDNA metabarcoding as a biomonitoring tool for marine protected
areas. PLoS One.

[B28] Hajibabaei M, deWaard JR, Ivanova NV, Ratnasingham S, Dooh RT, Kirk SL, Mackie PM, Hebert PD (2005). Critical factors for assembling a high volume of DNA
barcodes. Philos Trans R Soc Lond B Biol Sci.

[B29] Hebert PDN, Gregory TR (2005). The promise of DNA barcoding for taxonomy. Syst Biol.

[B30] Hebert PDN, Cywinska A, Ball SL, deWaard JR (2003). Biological identifications through DNA barcodes. Proc Biol Sci.

[B31] Hoffmann LS, Valdez F, Di Tullio J, Fruet P, Caon G, Boherer M, Freitas TRO (2008). Primeiro registro da presença de *Remora australis*
associada aos golfinhos nariz-de-garrafa, *Tursiops
truncatus*, nas águas do entorno do Arquipélago de São Pedro São
Paulo, Brasil.

[B32] Hubert N, Hanner R (2015). DNA Barcoding, species delineation and taxonomy: A historical
perspective. DNA Barcodes.

[B33] Ip YCA, Chang JJM, Lim KKP, Jaafar Z, Wainwright BJ, Huang D (2021). Seeing through sedimented waters: environmental DNA reduces the
phantom diversity of sharks and rays in turbid marine
habitats. BMC Ecol Evol.

[B34] Jarman SN, Berry O, Bunce M (2018). The value of environmental DNA biobanking for long-term
biomonitoring. Nat Ecol Evol.

[B35] Kimura M (1980). A simple method for estimating evolutionary rates of base
substitutions through comparative studies of nucleotide
sequences. J Mol Evol.

[B36] Knebelsberger T, Landi M, Neumann H, Kloppmann M, Sell AF, Campbell PD, Laakmann S, Raupach MJ, Carvalho GR, Costa FO (2014). A reliable DNA barcode reference library for the identification
of the North European shelf fish fauna. Mol Ecol Resour.

[B37] Krishnamurthy P, Francis RA (2012). A critical review on the utility of DNA barcoding in biodiversity
conservation. Biodivers Conserv.

[B38] Kumar S, Stecher G, Li M, Knyaz C, Tamura K (2018). MEGA X: Molecular evolutionary genetics analysis across computing
platforms. Mol Biol Evol.

[B39] Lakra WS, Verma MS, Goswami M, Lal KK, Mohindra V, Punia P, Gopalakrishnan A, Singh KV, Ward RD, Hebert P (2011). DNA barcoding Indian marine fishes. Mol Ecol Resour.

[B40] Limmon G, Delrieu-Trottin E, Patikawa J, Rijoly F, Dahruddin H, Busson F, Steinke D, Hubert N (2020). Assessing species diversity of Coral Triangle artisanal
fisheries: A DNA barcode reference library for the shore fishes retailed at
Ambon harbor (Indonesia). Ecol Evol.

[B41] Lin XL, Mo L, Bu WJ, Wang XH (2020). The first comprehensive DNA barcode reference library of Chinese
Tanytarsus (Diptera: Chironomidae) for environmental DNA
metabarcoding. Divers Distrib.

[B42] Lopez-Vaamonde C, Kirichenko N, Cama A, Doorenweerd C, Godfray HCJ, Guiguet A, Gomboc S, Huemer P, Landry JF, Laštůvka A (2021). Evaluating DNA barcoding for species identification and discovery
in European gracillariid moths. Front Ecol Evol.

[B43] Lubbock R, Edwards A (1981). The fishes of Saint Paul’s Rocks. J Fish Biol.

[B44] Ludt WB, Rocha LA (2015). Shifting seas: The impacts of Pleistocene sea-level fluctuations
on the evolution of tropical marine taxa. J Biogeogr.

[B45] Luiz OJ, Mendes TC, Barneche DR, Ferreira CGW, Noguchi R, Villaça RC, Rangel CA, Gasparini JL, Ferreira CEL (2015). Community structure of reef fishes on a remote oceanic island (St
Peter and St Paul’s Archipelago, equatorial Atlantic): The relative
influence of abiotic and biotic variables. Mar Freshw Res.

[B46] Lynggaard C, Bertelsen MF, Jensen CV, Johnson MS, Frøslev TG, Olsen MT, Bohmann K (2022). Airborne environmental DNA for terrestrial vertebrate community
monitoring. Curr Biol.

[B47] Mabragaña E, Delpiani SM, Rosso JJ, González-Castro M, Antoni MD, Hanner R, Astarloa JMD, Trivedi S, Ansari A, Ghosh S, Rehman H (2016). DNA barcoding in marine perspectives.

[B48] Macena BCL, Hazin FHV (2016). Whale shark (Rhincodon typus) seasonal occurrence, abundance and
demographic structure in the mid-equatorial atlantic ocean. PLoS One.

[B49] Magris RA, Pressey RL (2018). Marine protected areas: Just for show?. Science.

[B50] Maiello G, Talarico L, Carpentieri P, De Angelis F, Franceschini S, Harper LR, Neave EF, Rickards O, Sbrana A, Shum P (2022). Little samplers, big fleet: eDNA metabarcoding from commercial
trawlers enhances ocean monitoring. Fish Res.

[B51] McClenaghan B, Fahner N, Cote D, Chawarski J, McCarthy A, Rajabi H, Singer G, Hajibabaei M (2020). Harnessing the power of eDNA metabarcoding for the detection of
deep-sea fishes. PLoS One.

[B52] Mecklenburg CW, Møller PR, Steinke D (2010). Biodiversity of arctic marine fishes: Taxonomy and
zoogeography. Mar Biodiv.

[B53] Meierotto S, Sharkey MJ, Janzen DH, Hallwachs W, Hebert PDN, Chapman EG, Smith MA (2019). A revolutionary protocol to describe understudied hyperdiverse
taxa and overcome the taxonomic impediment. Mitt Mus Naturkunde Berl Dtsch Entomol Z.

[B54] Mendonça SA, Macena BCL, Afonso AS, Hazin FHV (2018). Seasonal aggregation and diel activity by the sicklefin devil ray
Mobula tarapacana off a small, equatorial outcrop of the Mid-Atlantic
Ridge. J Fish Biol.

[B55] Menezes NA, Buckup PA, Figueiredo JL, Moura RL (2003). Catálogo das espécies de peixes marinhos do Brasil.

[B56] Meusnier I, Singer GA, Landry J-F, Hickey DA, Hebert PD, Hajibabaei M (2008). A universal DNA mini-barcode for biodiversity
analysis. BMC Genomics.

[B57] Miya M, Sato Y, Fukunaga T, Sado T, Poulsen JY, Sato K, Minamoto T, Yamamoto S, Yamanaka H, Araki H I (2015). MiFish, a set of universal PCR primers for metabarcoding
environmental DNA from fishes: Detection of more than 230 subtropical marine
species. R Soc Open Sci.

[B58] Miya M (2022). Environmental DNA metabarcoding: A novel method for biodiversity
monitoring of marine fish communities. Ann Rev Mar Sci.

[B59] Mohriak WU (2020). Genesis and evolution of the South Atlantic volcanic islands
offshore Brazil. Geo-Mar Lett.

[B60] Moritz C, Cicero C (2004). DNA barcoding: Promise and pitfalls. PLoS Biol.

[B61] Nagarajan M, Parambath A, Prabhu V, Trivedi S, Rehman H, Saggu S, Panneerselvam C, Ghosh S (2020). DNA barcoding and molecular phylogeny.

[B62] Nelson JS, Grande TC, Wilson MV (2016). Fishes of the world.

[B63] Ogwang J, Bariche M, Bos AR (2020). Genetic diversity and phylogenetic relationships of threadfin
breams (Nemipterus spp.) from the Red Sea and eastern Mediterranean
Sea. Genome.

[B64] Pimentel CR, Andrades R, Ferreira CEL, Gadig OBF, Harvey ES, Joyeux J-C, Giarrizzo T (2020). BRUVS reveal locally extinct shark and the way for shark
monitoring in Brazilian oceanic islands. J Fish Biol.

[B65] Pinheiro HT, Bernardi G, Simon T, Joyeux JC, Macieira RM, Gasparini JL, Rocha C, Rocha LA (2017). Island biogeography of marine organisms. Nature.

[B66] Pinheiro HT, Macena BCL, Francini-Filho RB, Ferreira CEL, Albuquerque FV, Bezerra N, Carvalho-Filho A, Ferreira RCP, Luiz OJ, Mello TJ (2020). Fish biodiversity of Saint Peter and Saint Paul’s Archipelago,
Mid-Atlantic Ridge, Brazil: New records and a species
database. J Fish Biol.

[B67] Pinsky ML, Eikeset AM, McCauley DJ, Payne JL, Sunday JM (2019). Greater vulnerability to warming of marine versus terrestrial
ectotherms. Nature.

[B68] Porter T, Hajibabaei M (2020). Putting COI metabarcoding in context: The utility of Exact
Sequence Variants (ESVs) in biodiversity analysis. Front Ecol Evol.

[B69] Rathnasuriya MIG, Mateos-Rivera A, Bandara AGGC, Skern-Mauritzen R, Jayasnghe RPPK, Krakstad JO, Dalpadado P (2019). DNA barcoding confirms the first record of a Desmodema
polystictum (Ogilby, 1898) egg and all-time high adult catches in the Indian
Ocean. Mar Biodivers Rec.

[B70] Ribeiro AO, Caires RA, Mariguela TC, Pereira LHG, Hanner R, Oliveira C (2012). DNA barcodes identify marine fishes of São Paulo State,
Brazil. Mol Ecol Resour.

[B71] Rock J, Costa FO, Walker DI, North AW, Hutchinson WF, Carvalho GR (2008). DNA barcodes of fish of the Scotia Sea, Antarctica indicate
priority groups for taxonomic and systematics focus. Antarct Sci.

[B72] Russo T, Maiello G, Talarico L, Baillie C, Colosimo G, D’Andrea L, Di Maio F, Fiorentino F, Franceschini S, Garofalo G (2021). All is fish that comes to the net: Metabarcoding for rapid
fisheries catch assessment. Ecol Appl.

[B73] Schwentner M, Zahiri R, Yamamoto S, Husemann M, Kullmann B, Thiel R (2021). eDNA as a tool for non-invasive monitoring of the fauna of a
turbid, well-mixed system, the Elbe estuary in Germany. PLoS One.

[B74] Sharifuzzaman SM, Rasid MH, Rubby IA, Debnath SC, Xing B, Chen G, Chowdhury MSN, Hossain MS (2021). DNA barcoding confirms a new record of flyingfish Cheilopogon
spilonotopterus (Beloniformes: Exocoetidae) from the northern Bay of
Bengal. Conserv Genet Resour.

[B75] Shokralla S, Hellberg RS, Handy SM, King I, Hajibabaei M (2015). A DNA Mini-Barcoding system for authentication of processed fish
products. Sci Rep.

[B76] Singer GAC, Fahner NA, Barnes JG, McCarthy A, Hajibabaei M (2019). Comprehensive biodiversity analysis via ultra-deep patterned flow
cell technology: A case study of eDNA metabarcoding seawater. Sci Rep.

[B77] Soares MO, Lucas CC (2018). Towards large and remote protected areas in the South Atlantic
Ocean: St. Peter and St. Paul´s Archipelago and the Vitória-Trindade
Seamount Chain. Mar Policy.

[B78] Stat M, Huggett MJ, Bernasconi R, DiBattista JD, Berry TE, Newman SJ, Harvey ES, Bunce M (2017). Ecosystem biomonitoring with eDNA: Metabarcoding across the tree
of life in a tropical marine environment. Sci Rep.

[B79] Steinke D, Zemlak TS, Boutillier JA, Hebert PDN (2009). DNA barcoding of Pacific Canada’s fishes. Mar Biol.

[B80] Sultana S, Ali ME, Hossain MAM, Naquiah N, Zaidul ISM (2018). Universal mini COI barcode for the identification of fish species
in processed products. Food Res Int.

[B81] Taberlet P, Bonin A, Zinger L, Coissac E (2018). Environmental DNA: For biodiversity research and monitoring.

[B82] Taberlet P, Coissac E, Hajibabaei M, Rieseberg LH (2012). Environmental DNA. Mol Ecol.

[B83] Thomsen PF, Willerslev E (2015). Environmental DNA - an emerging tool in conservation for
monitoring past and present biodiversity. Biol Conserv.

[B84] Untergasser A, Cutcutache I, Koressaar T, Ye J, Faircloth BC, Remm M, Rozen SG (2012). Primer3--new capabilities and interfaces. Nucleic Acids Res.

[B85] Vaske TJ, Lessa RP, de Nóbrega M, Montealegre-Quijano S, Marcante SF, Bezerra JLJ (2005). A checklist of fishes from Saint Peter and Saint Paul
Archipelago, Brazil. J Appl Ichthyol.

[B86] Viana D, Hazin F, Souza MO (2009). Arquipélago de São Pedro e São Paulo: 10 anos de estação
científica.

[B87] Viana D, Hazin F, Andrade H, Nunes D, Viana D (2015). Fisheries in the Saint Peter and Saint Paul archipelago: 13 years
of monitoring. B Inst Pesca.

[B88] Wangensteen OS, Palacín C, Guardiola M, Turon X (2018). DNA metabarcoding of littoral hard-bottom communities: High
diversity and database gaps revealed by two molecular
markers. PeerJ.

[B89] Ward RD (2012). FISH-BOL, a case study for DNA barcodes. Methods Mol Biol.

[B90] Ward RD, Hanner R, Hebert PDN (2009). The campaign to DNA barcode all fishes, FISH-BOL. J Fish Biol.

[B91] Ward RD, Zemlak TS, Innes BH, Last PR, Hebert PDN (2005). DNA barcoding Australia’s fish species. Philos Trans R Soc Lond B Biol Sci.

[B92] Wilcox TM, McKelvey KS, Young MK, Jane SF, Lowe WH, Whiteley AR, Schwartz MK (2013). Robust detection of rare species using environmental DNA: The
importance of primer specificity. PLoS One.

[B93] Wingert N, Milmann L, Baumgarten M, Danilewicz D, Sazima I, Ott P (2021). Relationships between common Bottlenose Dolphins (Tursiops
truncatus) and Whalesuckers (Remora australis) at a remote archipelago in
the Equatorial Atlantic Ocean. Aquat Mamm.

[B94] Yu DW, Ji Y, Emerson BC, Wang X, Ye C, Yang C, Ding Z (2012). Biodiversity soup: Metabarcoding of arthropods for rapid
biodiversity assessment and biomonitoring. Methods Ecol Evol.

[B95] Zamani A, Fric ZF, Gante HF, Hopkins T, Orfinger AB, Scherz MD, Bartonová AS, Pos DD (2022). DNA barcodes on their own are not enough to describe a
species. Syst Entomol.

[B96] Zhang J, Hanner R (2011). DNA barcoding is a useful tool for the identification of marine
fishes from Japan. Biochem Syst Ecol.

